# Recent Histological Views as Regards Enamel and Dentine

**Published:** 1870-02

**Authors:** Henry R. Noel

**Affiliations:** Prof. of Physiology.


### AMERICAN JOURNAL

OF

### DENTAL SCIENCE.

Vol. III. THIRD SERIES--FEBRUARY 1870. No. 10.

### *Recent Histological Views as Regards Enamel and Dentine.*

An Abstract of a Lecture delivered Jan. 4th, 1870, at the Balto, College of Dent. Surg.

By Henry R. Noel, M.D., Prof. of Physiology.

Immediately after the closure of the follicles by the opercula and the inaugeration of the saccular stage, there begin certain changes which have an important bearing upon the further development of the tooth. The tunica propria now invests the papilla and the tunica reflexa lines the inner surface of the dental sac, and in the space between these, *i. e.* between papilla and inner surface of dental sac, we have a space in which *enamel, dentine, and cement, organs so called*, are to be developed. But just here a most thorough change takes place; the papilla begins to lose its character as a papilla and to acquire that of a pulp—vessels and immature connective tissue come into existence, and the vascularity is greatly increased. In the walls of the dental sac or capsule a similar change occurs, and we find that here also the vascularity is greatly increased; in fact, the blood vessels gradually extending invade the entire sac or capsule and lie directly beneath the outer membrane of the sac and directly above the tunica reflex, or perhaps more correctly, directly *external* to the tunica reflexa; the walls of the *sac* consisting now of two delicate membranes with a vascular net work between the two. The inner of these two membranes is the tunica reflexa, and between it and the pulp now vascular, we will suppose that nothing is found, and in this space we purpose to show you the progressive development and final calcification of the enamel and dentine organs.

Upon the *inner surface of tunica reflexa*, and fed by the vessels upon its external surface, we shall find masses of germinal matter; or nuclear matter, if you prefer the latter term; and these masses increasing in number grow inwards in the direction of the late papilla but now vascular pulp; simultaneously there is found upon the external surface of the pulp, *i. e.* external to and resting upon the tunica propria, masses of germinal matter; these two, different and distinct collections of germinal matter, fed by different and distinct vessels, increase rapidly in quantity, the first named collection growing inwards, and the second collection growing outwards, of course must grow towards each other and meet.

This line of union—this line of meeting shall hereafter mark the *line* of contact between the enamel, and the dentine; we will call this line for convenience sake "The Line of Conjunction"; it will be the boundary between enamel dentine.

Now a careful analysis of the subsequent changes, proves that upon this very line, the first definitely formed elements appear; or, in the language of Dr. L. S. Beale, the germinal matter changes to formed material and ceases to respond to the "carmine test," first at this line of junction; here we have a determinate tissue—a definite anatomical element arising, and this tissue or these anatomical forms increase in quantity, but they increase in exactly an opposite direction to the growth of the germinal matter.

The germinal matter begins on the internal surface of the dental capsule for enamel, and upon the external surface of the pulp for dentine; the two growing, the one inwards and the other outwards, meet at the "*line of conjunction;*" the enamel organ growing in, the dentine organ growing out as regards germinal matter, but as regards the formed material or first definitely formed elements, we find them beginning at the *line* and extending outwards for enamel and inwards for dentine. The formed material for enamel and the formed material for dentine. begin first at this *line*,—first at a common starting point and grow or increase in diametrically opposite directions; that for the enamel grows out towards the dental sac—that for dentine grows in towards the pulp —as a necessity, therefore, for each organ the formed material grows back along the exact line through which the germinal matter advanced.

The "formed material," or anatomical forms, the terms meaning exactly the same thing, will finally reach the dental sac for enamel—and reach the dental pulp for dentine; the enamel organ is now fully formed and lies between *capsule* or *sac* and "*Line of Conjunction*"; the dentine organ is also now fully formed, and lies between the *pulp* and "*line of conjunction*"; of course upon this *line* the two *organs* meet and touch each other.

Now as to the constituent elements of the two organs, what are their shapes, or characteristic forms respectively?

Enamel Organ—Is composed of little bodies resembling rods, or columns, these little bodies are placed side by side and packed closely together, so very closely are they packed that they compress each other and from being more or less cylindrical they become somewhat hexagnonal or at any rate polygonal; these bodies extend from "line of conjunction" to the inner surface of the dental sac, and have no intervening substance whatever, but each rests freely and fully against its neighbors; many of the bodies contain nuclei, especially near the ends in contact with the sac, these nuclei are nothing more than masses of germinal matter which have not as yet changed to formed material, but most of them are changing, and [illegible]l should ultimately change into it.

These enamel rods or columns, are first found upon the "line of conjunction," and can be traced by progressive steps, from this line out to the inner surface of the sac; here we notice, that the germinal matter is *all* consumed in the production of these columns or little bodies, and the space between the *sac* and line of conjunction, once occupied by germinal matter, is now occupied by formed material in the shape of closely packed rods, columns or bodies. This is the *enamel's organic Basis*, and when this becomes calcified we have *enamel.*

Dentine Organ. From the "line of conjunction," to the pulp proper, we have the dentine organ, and the statement made as regards enamel is also true as regards dentine; viz; that the formed material begins on the line of conjunction and extends to the pulp, but here there is a slight difference as regards germinal matter; for in the enamel organs every atom of germinal matter changes to formed material, every atom is or should be consumed in the production of the soft, solid columns or rods of which the enamel organ is composed, but this is not so comprehensively true of the dentine organ, all of the germinal matter is *not* changed into the formed material, a *portion* remains unchanged.

The dentine organ, therefore, will exhibit under the microscope and under the "*carmine test*" of Dr. L. S. Beale, both formed material and germinal matter, the formed material being found in the shape of "Columnar Epithelial Cells Nucleated" and the germinal matter being found as the *nuclei of these cells.*

A critical analysis of the dentine organ under the microscope, shows the organ to be composed entirely of these cell elements, or columnar bodies and the nuclear matter, so arranged as to give a fibrillated appearance to the whole organ; the cells or bodies are extremely minute, in fact they are not *true cells* as they have no distinct walls, but appear to be solid cylinders with a central portion of nuclear matter, and are arranged in linear series so as to form *Fibrillæ;* the whole organ just before calcification, consists of these fibrillæ and there is *no* interfibrillar substance whatever. In many and perhaps in most of the fibrillæ at this period, there is a thorough conversion of the central axis of nuclear matter into formed material, or into solid fibril; these solid fibrils, then run from line of conjunction to pulp, and consist entirely of formed material, but there are many that run between the same points, which still retain a central axis of nuclear matter, having only their periphery of formed material.

Now these two kinds of fibrillæ lie side by side, and represent different stages only, not different kinds of tissue; the tissue is identically the same in each kind, the only distinction being that in the one the whole of the germinal matter has changed to formed material, in the other a central portion remains unchanged; the shape &c., of the two are the same, their whole nature and their characteristics identical.

The dentine organ, therefore consists of nothing but fibrillæ, some of them solid (completely changed throughout) others apparently solid but having in reality a central axis of nuclear matter, which may or may not change to formed material, though liable to and capable of doing so at any time; should this central portion, of nuclear matter, change to formed material, the fibril would be solid and would then be identical with the other solid fibrils.

Relations to the Pulp.—The fibrils run into and blend in organic continuity with the tissue on the peripheral surface of the pulp; on this peripheral surface we find the same columnar nucleated bodies and nuclear matter; the connectissue corpuscles and nuclear cylinders of the incompletely changed fibrillæ run together, and the tissue and the solid fibrillar substance blend in organic continuity.

The dentine organ extends, therefore, from the line of conjunction to the pulp, and rest upon this pulp, directly and immediately, with no tissue or structure between organ and pulp, save where immature connective tissue crops out here or there; even then the organ and this tissue blend so intimately that it is impossible to make any distinction in their anatomical elements.

Calcification.—The enamel organ being formed and the dentine organ being formed, calcification begins on the line of conjunction and extends out towards the *sac* for enamel, and in towards the pulp for dentine ; the calcification is thorough and complete for enamel—all of the polygonal bodies being completely calcified. For dentine the calcification is *not so very thorough and complete*; the solid fibrillæ are calcified thoroughly, but many of those having central masses of nuclear matter are only partially calcified, leaving the central nuclear matter, and more or less of fibril surrounding it still uncalcified. The calcification is more thorough near the periphery and less complete as we approach the pulp, hence the uncalcified fibrillæ are very small indeed near the line of conjunction but large near the pulp.

The calcified enamel organ gives us the enamel, the shape and general outline of the rods or bodies remaining the same ; the calcified dentine organ gives us dentine, but the calcification is complete in the former and partial or incomplete in the latter, and the incomplete calcification gives rise to the "fleshy fibrillæ," found in the dentine of the fresh tooth, or in recently extracted teeth.

On the line of union of enamel and dentine, there is an almost complete calcification of the fibrillæ, the portions remaining uncalcified are so exceedingly minute that they appear as the faintest of thread like traces, but from this line towards the pulp the calcification appears to involve less and less of the fibrils until we reach the pulp itself and here the fibrils are of some considerable size.

The calcified fibrils form dentine, those uncalcified retain their character as fibrils ; but, and I wish to be clearly comprehended upon this point, those which remain uncalcified during the first processes of calcification, and even after the tooth is cut and has passed through the gum, *may become calcified at some subsequent period* and form dentine. It is probable that after the teeth are cut, a slow but progressive calcification takes place until the death of the individual or death of the pulp.

Dentine will thus become *denser* or more condensed as the tooth becomes older, and that portion of the dentine organ which is left uncalcified around the pulp, may also become calcified to a greater or less extent, and the dentine be increased in thickness from pulp towards the enamel. In fact the dentine organ, so called, may even after adult life increase, and encroach upon the pulp cavity and becoming calcified diminish the size of that cavity. The fibrils prior to calcification, are not simply straight, but they curve, wave, divide and branch ; and as this is true of the " whole organ," it will be true of that portion which remaining uncalcified, we have to study as dentinal fibrillæ.

From the above statements we deduce these conclusions :

I.—That the dentinal organ is composed of fibrillæ, more or less aborescent or branching.

II.—The organ extends to the pulp, and surrounds and closely invests the pulp.

III.—The calcification of a portion of the Fibrillæ gives us hard dentine.

IV.—The portion remaining uncalcified, we call dentinal fibrillæ.

V.—That the dentinal fibrillæ are liable at any time to become more or less calcified and form dentine.

VI.—That there is no difference between the calcfied and the uncalcified fibrillæ, unless we except the central axis of nuclear matter as distinctive.

VII.—That this central axis is liable at any time to change into fibril, and, as fibril, liable to be calcifiied, and form dentine.

VIII.—There is no inter-fibrillar substance whatever.

IX.—Fresh dentine, therefore, has no tubuli but has uncalcified fibrillæ, solid or nearly solid.

X.—That the tubuli found in the dry tooth, or dead and dry tooth, and formed by the dessication are evaporation of these dentinal fibrillæ, and have no existence in the living tooth.

XI.—That the difference between dentine and the fibrillæ is simply one of mineral element, and the fibrillæ may at any time take up this mineral element and become true dentine, *i. e. calcify.*

XII.—Enamel and dentine are not vitalized tissues, as far as the calcified portions are concerned; enamel is dead—perfectly dead—and dentine would be equally as much so, were it not for the fibrillæ uncalcified; enamel proper and dentine proper are amenable to physical and chemical laws only, and therefore any sensitiveness in dentine is in virtue of its fibrillæ, and of their organic continuity with the terminal nerve fibres in the pulp.

---

